# Impact of hydrophilic substances on Ostwald ripening in emulsions stabilized by varied hydrophilic group surfactants

**DOI:** 10.1038/s41538-024-00316-4

**Published:** 2024-10-05

**Authors:** Jihyeon Kim, Yejin Noh, David Julian McClements, Seung Jun Choi

**Affiliations:** 1 Department of Food Science and Biotechnology, Seoul National University of Science and Technology, Seoul, Republic of Korea; 2 Department of Food Science, University of Massachusetts, Amherst, MA USA; 3 Center of Functional Biomaterials, Seoul National University of Science and Technology, Seoul, Republic of Korea

**Keywords:** Nutrition, Colloids

## Abstract

This study investigated the impact of water-soluble substances on Ostwald ripening in emulsions stabilized by surfactants with different head groups (Brij S20 and Tween 60). Adding ≥20% (*w*/*w*) corn oil to the oil phase effectively inhibited Ostwald ripening of *n*-decane emulsions due to compositional ripening. The presence of glucose, maltose, or glycerol in the aqueous phase of the emulsions decreased the Ostwald ripening rate, regardless of emulsifier type. However, the impact of propylene glycol depended on emulsifier type, accelerating Ostwald ripening in Brij S20-stabilized emulsions but having little effect in Tween 60-stabilized emulsions. This effect was mainly attributed to the ability of propylene glycol to alter interfacial characteristics. When emulsions were fabricated with a mixture of *n*-decane and corn oil, glucose and maltose were still effective in inhibiting Ostwald ripening, but glycerol lost its ability. These results have important implications for formulating emulsion-based delivery systems with enhanced shelf life.

## Introduction

The lipids in many processed foods are present either partly or wholly in an emulsified form^1,2^. The most common type of emulsified lipids in the food industry are oil-in-water emulsions, which consist of oil droplets dispersed in water. Since water and oil are immiscible, emulsifiers are needed to stabilize these systems. These emulsifiers facilitate the formation of emulsions, as well as inhibiting their breakdown, by creating a protective coating around the oil droplets^3,4^. Nevertheless, emulsions are thermodynamically unstable systems that will breakdown due to several destabilization physicochemical mechanisms, including creaming, flocculation, coalescence, phase inversion, and Ostwald ripening^2^. These instability mechanisms lead to alterations in the size and location of the oil droplets in the emulsions, which can reduce the shelf life of the end product.

In this study, we focused on the impact of Ostwald ripening (OR) on the stability of oil-in-water emulsions. This instability mechanism plays an important role in emulsions that contain oil phases that have a relatively high solubility in water^5–7^. In the food industry, this includes essential oils^8,9^, flavor oils^10–12^, and shorter chain triacylglycerol oils^13,14^. As the size of the oil droplets in emulsions decreases, their curvature increases, which enhances the solubility of the oil phase in the surrounding aqueous phase. Therefore, the OR rate is typically higher in nanoemulsions than in the equivalent macroemulsions^15^. When formulating emulsions, particularly nanoemulsions, using oils that have an appreciable water solubility, it is therefore important to account for Ostwald ripening.

In oil-in-water emulsions, Ostwald ripening results in a net increase in average droplet size due to the transport of oil molecules through the aqueous phase from the smaller oil droplets to the larger ones^2,16^. In practice, Ostwald ripening can also accelerate the breakdown of emulsions by increasing the rate of other instability mechanisms such as coalescence and creaming. This is because larger oil droplets are typically more susceptible to coalescence and creaming than smaller ones.

A practical means of retarding Ostwald ripening in emulsions containing relatively polar oils is to incorporate ripening inhibitors^9,17^. These are hydrophobic water-insoluble substances that are mixed with water-soluble polar oils prior to emulsion fabrication. Ripening inhibitors retard or prevent droplet growth due to an entropy of mixing effect that counterbalances the interfacial curvature effect that normally drives Ostwald ripening^5,16^. Another advantage of using ripening inhibitors is that they may also act as weighting agents that retard droplet creaming. Triglycerides, the most common ripening agent used in the food industry, have a higher density than most polar oils (like essential or flavor oils), and so the density contrast between the oil and water phases is reduced, thereby retarding creaming^18^.

The rate of Ostwald ripening in emulsions containing a pure oil phase can be described by the following equation:

1 $${d(t)}^{3}={d(0)}^{3}+\frac{64\gamma {V}_{\!M}{DSt}}{9{RT}}$$

Here, *d*(t) is the diameter at time *t*, γ is the oil-water interfacial tension, *V*_M_ is the molar volume of the oil phase, *D* is the diffusion coefficient of the oil molecules through the water phase, *S* is the equilibrium solubility of the oil in the water phase, *R* is the gas constant, and *T* is the absolute temperature. This equation predicts that droplet growth due to Ostwald ripening should increase as the solubility and diffusion of the oil molecules in the water phase increases, as well as when the interfacial tension increases. Consequently, emulsifiers may influence Ostwald ripening by altering interfacial tension, whereas aqueous phase additives may impact Ostwald ripening by altering the viscosity of the continuous phase, thereby altering the diffusion coefficient (which is inversely proportional to the viscosity). For this reason, we examined the impact of emulsifier type and aqueous phase additives on the resistance of model oil-in-water emulsions to Ostwald ripening.

In practical applications, beverage emulsions are usually fabricated in a concentrated form and then diluted during the preparation of the final product^19^. Consequently, water-soluble additives such as sweeteners and stabilizers may be present at relatively high levels in the aqueous phase of concentrated beverage emulsions. Therefore, we examined the impact of these kinds of water-soluble additives on the OR stability of oil-in-water emulsions containing a model oil (*n*-decane) with an appreciable water-solubility. The knowledge gained in this study may help to optimize the formulation of more stable emulsions for application in the food and other industries.

## Results and discussion

### Influence of surfactant type on Ostwald ripening

Initially, the impact of the structures of the small molecule surfactants on Ostwald ripening was studied by using Tween 60 and Brij S20 as model surfactants. The hydrophobic groups on the two surfactants were similar (C_18_ alkane chains) but the hydrophilic groups were different. Both surfactants have hydrophilic groups consisting of polyoxyethylene chains with the same overall number of monomers. But Tween 60 has three shorter polyoxyethylene chains, whereas Brij S20 has one long one. Consequently, Brij S20 would be expected to form thicker and denser interfacial layers than Tween 60. Moreover, this difference may have led to differences in the interfacial tension and mass transport processes in the emulsions. Since the hydrophobic tails of the two surfactants were similar, but their hydrophilic heads were different, we expected them to behave differently.

**Fig. 1:**
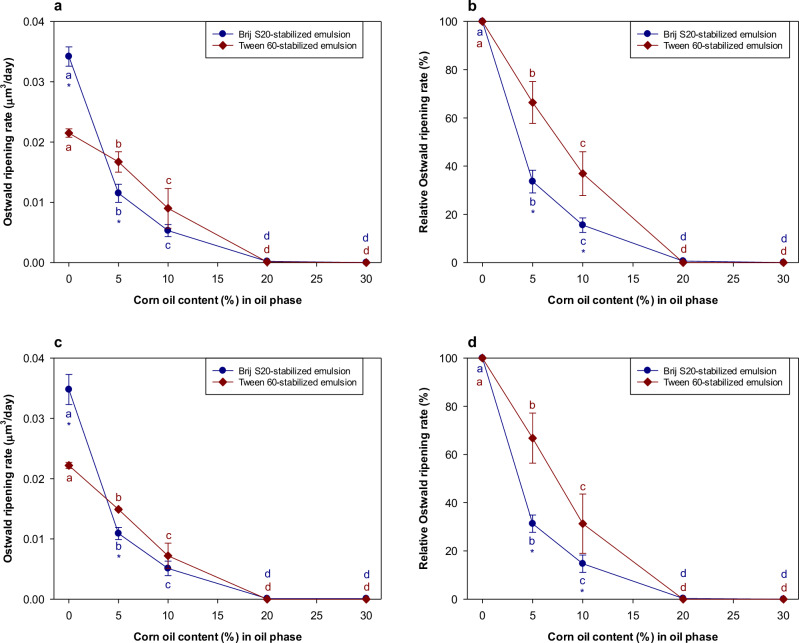
Influence of the modification of oil composition by mixing corn oil on the Ostwald ripening rates of emulsions stored at different pH values. **a** Ostwald ripening rates of Brij S20- and Tween 60-stabiilzed emulsions stored at pH 3. **b** Relative Ostwald ripening rates of Brij S20- and Tween 60-stabiilzed emulsions stored at pH 3. **c** Ostwald ripening rates of Brij S20- and Tween 60-stabiilzed emulsions stored at pH 7. **d** Relative Ostwald ripening rates of Brij S20- and Tween 60-stabiilzed emulsions stored at pH 7. Values denoted by the different letters indicate the significant differences within emulsions prepared with the same emulsifier (*p* ≤ 0.05). Asterisk (*) indicates the significant difference between Brij S20- and Tween 60-stabilized emulsions containing the same amount of corn oil (*p* ≤ 0.05).

The droplet growth rates of Tween 60- and Brij S20-stabilized emulsions were measured and compared (Fig. 1). These experiments were carried out under acidic (pH 3) and neutral (pH 7) conditions because this covers the pH range typically found in commercial beverage products. For both surfactants, solution pH did not have a major impact on the OR rate (Supplementary Fig. S1a), which may have been because Tween 60 and Brij S20 were both non-ionic surfactants, so pH did not strongly influence their interfacial properties. The droplet growth rates for the two emulsions were fairly similar during the early stages of storage (≤9 days) but then the oil droplets in the Brij S20-stabilized emulsions grew faster than those in the Tween 60-stabilized emulsions at longer times.

**Fig. 2:**
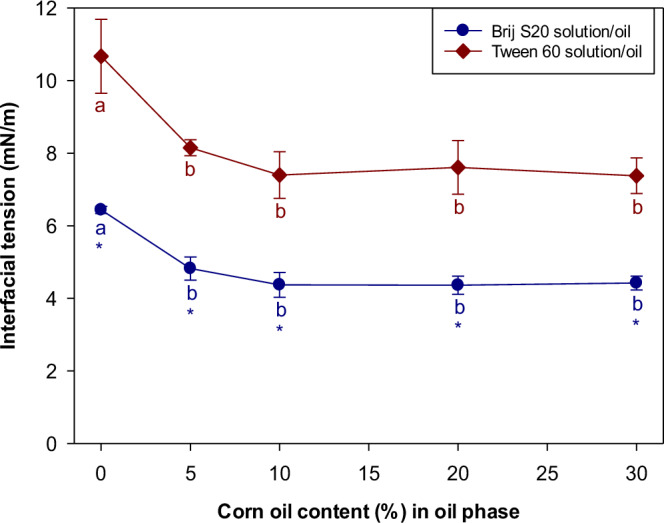
Influence of the corn oil addition on the interfacial tensions between oil and emulsifier solution. Values denoted by the different letters indicate the significant differences within the interfacial films prepared with the same emulsifier (*p* ≤ 0.05). Asterisk (*) indicates the significant difference between *n*-decane/Brij S20 solution and *n*-decane/Tween 60 solution when the corn oil content in the oil phase was same (*p* ≤ 0.05).

As shown in Eq. 1, the OR rate is predicted to increase with increasing interfacial tension. For this reason, the interfacial tensions of *n*-decane/surfactant solutions were measured (Fig. 2). The interfacial tension of the *n*-decane/Tween 60 solution was approximately 1.7 times higher than that of the *n*-decane/Brij S20 solution, which can be attributed to the fact that the Tween 60 molecules cannot pack as densely at the oil-water interface as the Brij S20 ones. Interfacial tension effects therefore do not account for the observed faster OR rate for the Tween 60-stabilized emulsions, as Eq. 1 predicts that the OR rate should increase with increasing interfacial tension.

### Influence of surfactants on the ability of ripening inhibitors in *n*-decane emulsions

The rate of Ostwald ripening in the emulsions was retarded by adding different amounts of a ripening inhibitor (corn oil) to the *n*-decane prior to homogenization. Previous studies have shown that water-insoluble oils (generally vegetable oils) are highly effective at inhibiting Ostwald ripening in emulsions, provided their concentration exceeds a particular level^12,20^. When the oil phase of an oil-in-water emulsion is comprised of both a water-soluble and a water-insoluble oil, the water-soluble oil can be transferred from the smaller to the larger oil droplets, whereas the water-insoluble oil cannot. After a certain period, the percentage of water-insoluble oil in the small droplets is greater than in the large droplets, which is thermodynamically unfavorable because an even distribution of the two oils throughout all of the oil phase is more entropically favorable^2^. The magnitude of this effect depends on the concentration difference between the small and large droplets, which depends on the initial concentration of the water-soluble and water-insoluble oils inside the droplets. Thus, a compositional ripening (CR) effect occurs that wants to drive the water-soluble oil from the large droplets to the small droplets to balance their compositions. At a sufficiently high ripening inhibitor concentration, the CR effect balances the Ostwald ripening effect, which prevents droplet growth.

As shown in Fig. 1 and Supplementary Fig. S2, the rate of droplet growth decreases as the corn oil concentration in the oil phase increased, which can be attributed to this CR effect. When 5 or 10% (*w*/*w*) corn oil were added to the oil phase, droplet growth was still observed in both emulsions during storage (Supplementary Fig. S1b, c), indicating that these levels of corn oil were not enough to completely inhibit Ostwald ripening. However, little or no droplet growth was observed in emulsions when the corn oil concentration in the oil phase was ≥ 20% (*w*/*w*) (Supplementary Fig. S1d, e). This finding suggested that 20% corn oil was sufficient to prevent Ostwald ripening in *n*-decane-in-water emulsions, independent of emulsifier type.

The following equations can be used to predict the initial mole fraction of the ripening inhibitor (*X*_2_) required to inhibit Ostwald ripening, either kinetically or thermodynamically^2^:

2 $$Unstable\,regime:{X}_{2} < \frac{2{\alpha }_{1}}{3{d}_{0}}$$

3 $$Kinetically\,stable\,regime:\frac{2{\alpha }_{1}}{3{d}_{0}} < {X}_{2} < \frac{2{\alpha }_{1}}{{d}_{0}}$$

4 $$Thermodynamically\,stable\,regime:{X}_{2} > \frac{2{\alpha }_{1}}{{d}_{0}}$$

Here, α_1_ = 2γ*V*_m_/*RT*, where *γ* is the interfacial tension (mN m^−1^), *V*_m_ is the molar volume of the ripening inhibitor, *R* (8.31 J mol^−1^ K^−1^) is the Universal gas constant, *T* is the absolute temperature (300 K), and *d*_0_ is the initial mean droplet diameter (0.450 and 0.442 μm for Brij S20- and Tween 60-stabilized emulsions, respectively). This equation therefore predicts that the mole fraction (*X*_2_) of corn oil required to inhibit Oswald ripening should be around 0.007 for kinetic stability or 0.022 for thermodynamic stability. The molar volume of the corn oil (9.5 × 10^−4^ m3 mol^−1^) was calculated as *V*_m_ = *M*_w_/*ρ*, where *M*_w_ is the molar mass (0.872 kg/mol) and *ρ* is the density (920 kg m^−3^) of the corn oil. In this study, the molar masses of the *n*-decane and corn oil were 143 and 872 g/mol, respectively. Experimentally, we found that the addition of 20% corn oil to the oil phase of the emulsions led to suppression of Oswald ripening (Fig. 1). This corresponds to a mole fraction (*X*_2_) of (20/872)/(20/872 + 80/143), i.e., 0.039. Thus, the experimental value of the amount of corn oil required to completely inhibit Oswald ripening is greater than the predicted value for thermodynamic stability.

The above equations indicate that the initial droplet diameter (*d*_0_) should influence the droplet growth rate^5^. Consequently, the impact of corn oil content in the oil phase on the initial droplet characteristics was determined (Supplementary Fig. S3). Regardless of surfactant type, the mean droplet diameter and polydispersity index of the freshly prepared emulsions did not depend strongly on oil phase composition. This effect would account for the fact that a similar amount of corn oil was required to retard droplet growth in all the emulsions.

### Influence of water-soluble additives on droplet growth rate without ripening inhibitors

As mentioned earlier, commercial food and beverage emulsions may contain various kinds of hydrophilic additives in their aqueous phases. For this reason, we investigated the effects of several water-soluble additives on Ostwald ripening of the *n*-decane oil-in-water emulsions: glucose, maltose, glycerol, and propylene glycol. These experiments were conducted at pH 3, since many commercial emulsified foods and beverages are acidic (like soft drinks). These experiments were initially conducted in the absence of corn oil to determine if the aqueous phase additives could be used to replace hydrophobic ripening inhibitors.

**Fig. 3:**
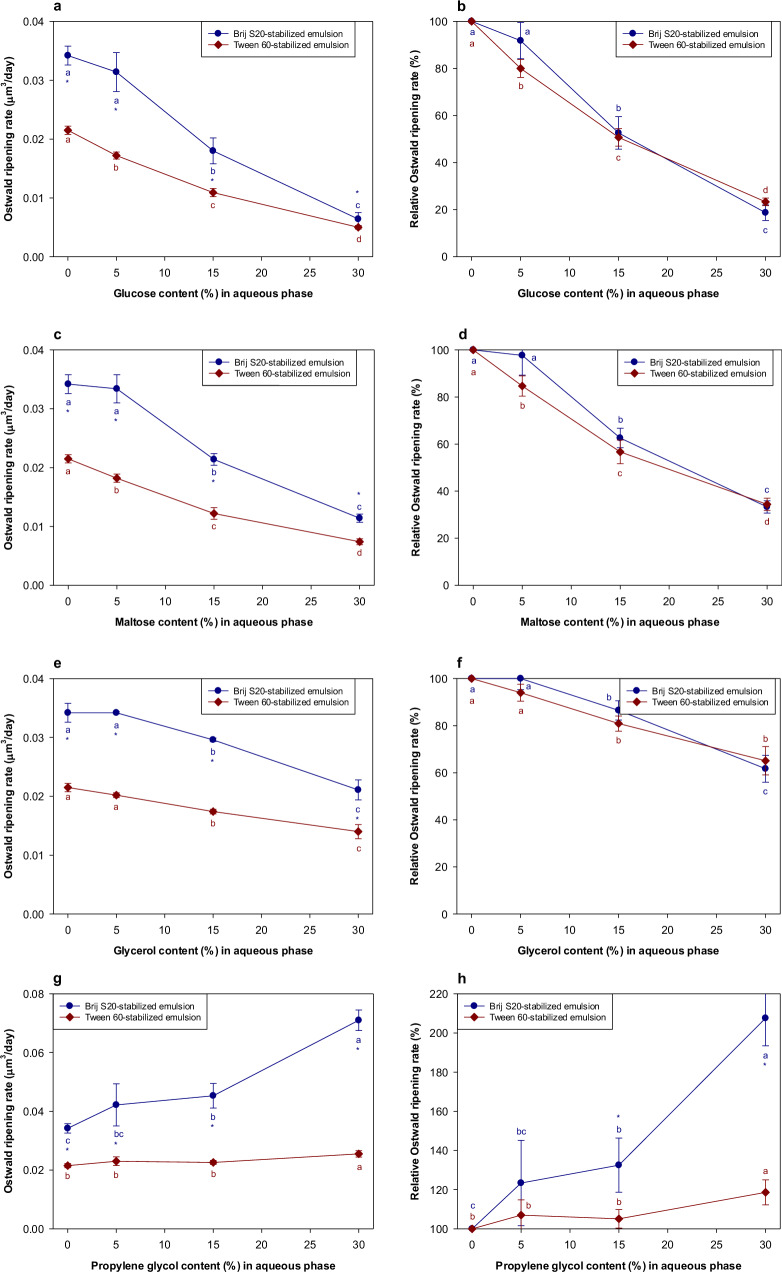
Influence of the water-soluble substances on the Ostwald ripening rates of emulsions prepared with the pure *n*-decane. **a** Ostwald ripening rates of Brij S20- and Tween 60-stabiilzed emulsions containing glucose. **b** Relative Ostwald ripening rates of Brij S20- and Tween 60-stabiilzed emulsions containing glucose. **c** Ostwald ripening rates of Brij S20- and Tween 60-stabiilzed emulsions containing maltose. **d** Relative Ostwald ripening rates of Brij S20- and Tween 60-stabiilzed emulsions containing maltose. **e** Ostwald ripening rates of Brij S20- and Tween 60-stabiilzed emulsions containing glycerol. **f** Relative Ostwald ripening rates of Brij S20- and Tween 60-stabiilzed emulsions containing glycerol. **g** Ostwald ripening rates of Brij S20- and Tween 60-stabiilzed emulsions containing propylene glycol. **h** Relative Ostwald ripening rates of Brij S20- and Tween 60-stabiilzed emulsions containing propylene glycol. Values denoted by the different letters indicate the significant differences within emulsions prepared with the same emulsifier (*p* ≤ 0.05). Asterisk (*) indicates the significant difference between Brij S20- and Tween 60-stabilized emulsions containing the same amount of water-soluble substance (*p* ≤ 0.05).

Glucose, maltose, and glycerol all inhibited Ostwald ripening in the emulsions, with the magnitude of the effect increasing with additive concentration (Fig. 3 and Supplementary Fig. S4). The results for propylene glycol are discussed later because it behaved differently to the other additives. Glucose and maltose exhibited similar OR-inhibiting effects, but glycerol was less effective. We therefore tried to establish the origin of this difference. There was no correspondence between the initial droplet size and polydispersity of the emulsions and the OR rate for these systems (Supplementary Fig. S5). Water-soluble additives may impact Ostwald ripening through several physicochemical mechanisms. According to Eq. 1, the OR rate should decrease as the interfacial tension decreases, the diffusion coefficient decreases (viscosity increases), and the equilibrium solubility of the oil in the aqueous phase decreases. Adding water-soluble additives to the aqueous phase may have impacted each of these factors.

The impact of each of the water-soluble additives on the viscosity of the aqueous phase was measured (Supplementary Fig. S6). At a given concentration, the solutions containing glucose and maltose had similar viscosities, which were higher than those of glycerol. Consequently, the reduced OR rate at higher additive concentrations could be due to their ability to increase solution viscosity, and thereby retard the diffusion of the oil molecules through the aqueous phase. Moreover, increasing the aqueous phase viscosity may have reduced the droplet-droplet collision frequency, thereby reducing oil transfer and coalescence of the droplets^2^.

**Fig. 4:**
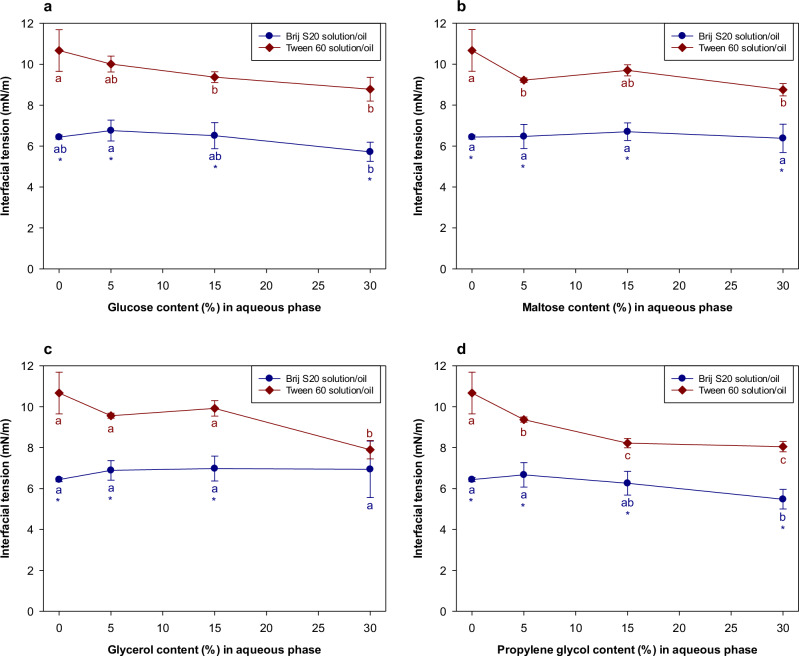
Influence of the addition of water-soluble substances on the interfacial tensions between *n*-decane and emulsifier solution. **a** Interfacial tension between *n*-decane/emulsifier solution containing glucose. **b** Interfacial tension between *n*-decane/emulsifier solution containing maltose. **c** Interfacial tension between *n*-decane/emulsifier solution containing glycerol. **d** Interfacial tension between *n*-decane/emulsifier solution containing propylene glycol. Values denoted by the different letters indicate the significant differences within the interfacial films prepared with the same emulsifier (*p* ≤ 0.05). Asterisk (*) indicates the significant difference between *n*-decane/Brij S20 solution and *n*-decane/Tween 60 solution when the content of water-soluble substance in aqueous phase was same (*p* ≤ 0.05).

The influence of the water-soluble additives on the interfacial tensions of the oil-water interface was also measured (Fig. 4a–c). For Tween 60, the oil-water interfacial tension gradually decreased with increasing additive concentration, regardless of additive type. This decrease in interfacial tension may therefore also have contributed to some extent to the observed decrease in the OR rate for Tween 60-stabilized emulsions. In contrast, for Brij S20, the water-soluble additives had little impact on the interfacial tension and so would not be expected to influence the OR rate of the emulsions. The different behavior of the two surfactants may have been because the additives were able to penetrate more easily into the less dense Tween 60 interfacial layers than the denser Brij S20 ones. As a result, the additives may have been able to alter the packing of the surfactants within the interfacial layer formed by Tween 60, thereby altering the interfacial tension, but less so for the Brij S20^21^. The reduction in OR rate of Tween 60- and Brij S20-stabilized emulsions cannot be easily generalized as being caused by changes in interfacial tension due to the addition of water-soluble substances. Other factors must also be important, such as the increase in the viscosity of the aqueous phase caused by the addition of the water-soluble substances or changes in interfacial structure.

### Influence of water-soluble additives on droplet growth rate with ripening inhibitors

**Fig. 5:**
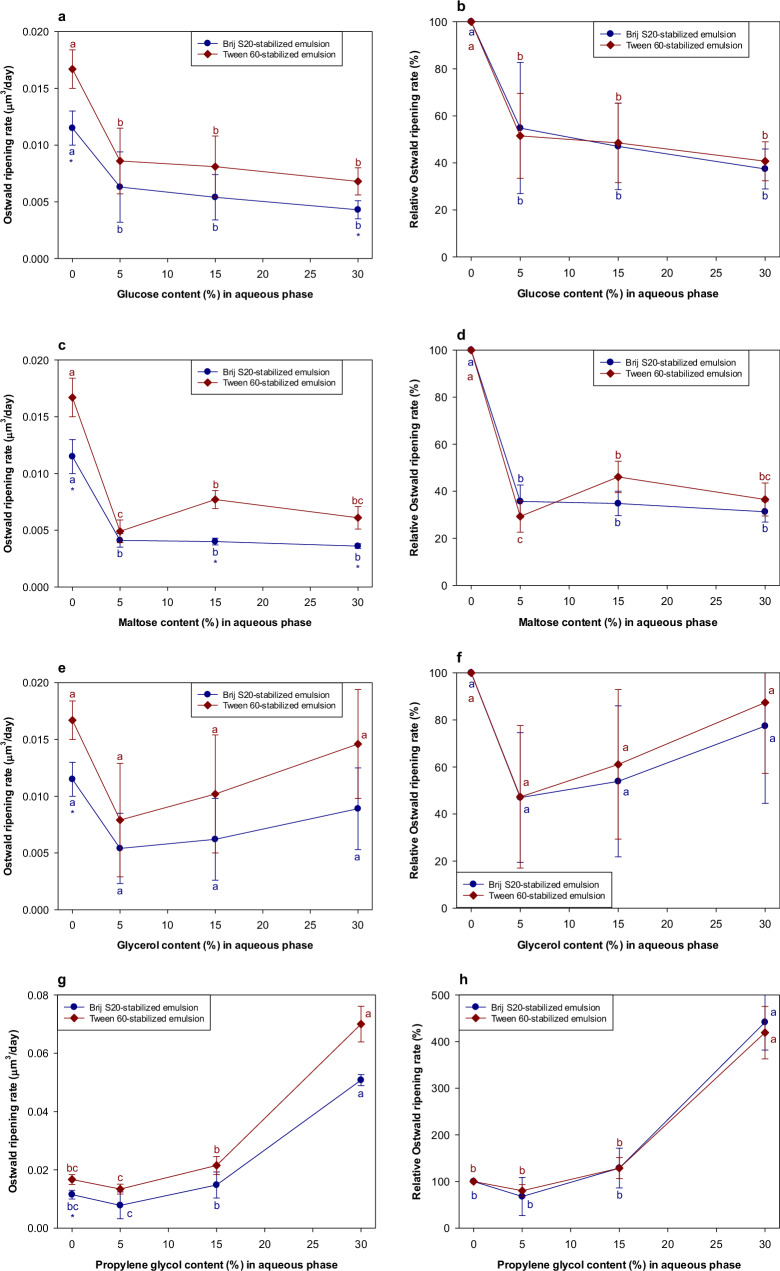
Influence of the water-soluble substances on the Ostwald ripening rates of emulsions prepared with the mixture of *n*-decane (95% (*w*/*w*)) and corn oil (5% (*w*/*w*)). **a** Ostwald ripening rates of Brij S20- and Tween 60-stabiilzed emulsions containing glucose. **b** Relative Ostwald ripening rates of Brij S20- and Tween 60-stabiilzed emulsions containing glucose. **c** Ostwald ripening rates of Brij S20- and Tween 60-stabiilzed emulsions containing maltose. **d** Relative Ostwald ripening rates of Brij S20- and Tween 60-stabiilzed emulsions containing maltose. **e** Ostwald ripening rates of Brij S20- and Tween 60-stabiilzed emulsions containing glycerol. **f** Relative Ostwald ripening rates of Brij S20- and Tween 60-stabiilzed emulsions containing glycerol. **g** Ostwald ripening rates of Brij S20- and Tween 60-stabiilzed emulsions containing propylene glycol. **h** Relative Ostwald ripening rates of Brij S20- and Tween 60-stabiilzed emulsions containing propylene glycol. Values denoted by the different letters indicate the significant differences within emulsions prepared with the same emulsifier (*p* ≤ 0.05). Asterisk (*) indicates the significant difference between Brij S20- and Tween 60-stabilized emulsions containing the same amount of water-soluble substance (*p* ≤ 0.05).

The water-soluble additives were unable to completely inhibit Ostwald ripening. For this reason, we examined the impact of combining these additives with a ripening inhibitor (corn oil) on the overall OR rate in the two types of emulsion. We hypothesized that using a combination of additives would be more effective than using a single one. To verify this hypothesis, the influence of water-soluble additive concentration on the OR rates of emulsions prepared containing 95% (*w*/*w*) *n*-decane and 5% (*w*/*w*) corn oil was evaluated (Fig. 5 and Supplementary Fig. S7).

**Fig. 6:**
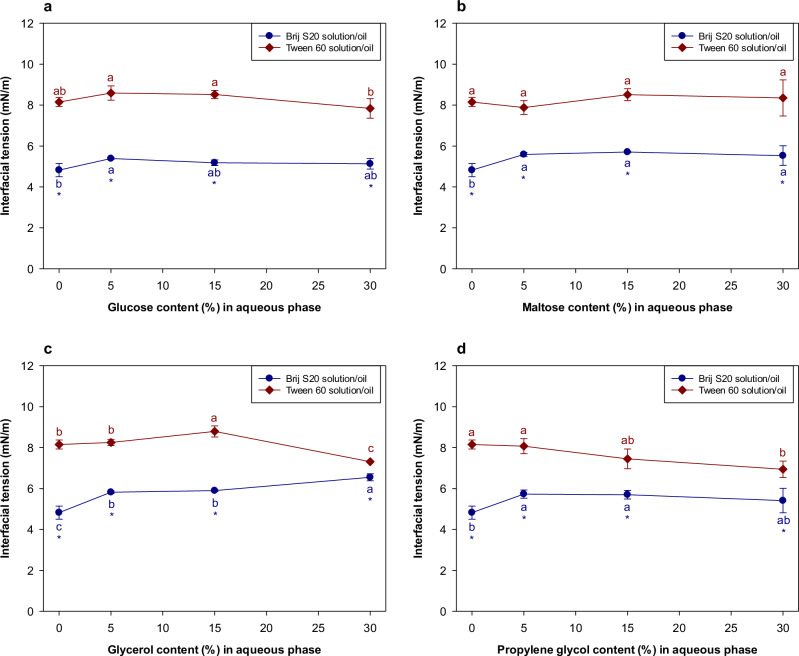
Influence of the addition of water-soluble substances on the interfacial tensions between oil (*n*-decane:corn oil = 95:5 (*w*/*w*)) and emulsifier solution. **a** Interfacial tension between *n*-decane/emulsifier solution containing glucose. **b** Interfacial tension between *n*-decane/emulsifier solution containing maltose. **c** Interfacial tension between *n*-decane/emulsifier solution containing glycerol. **d** Interfacial tension between *n*-decane/emulsifier solution containing propylene glycol. Values denoted by the different letters indicate the significant differences within the interfacial films prepared with the same emulsifier (*p* ≤ 0.05). Asterisk (*) indicates the significant difference between *n*-decane/Brij S20 solution and *n*-decane/Tween 60 solution when the content of water-soluble substance in aqueous phase was same (*p* ≤ 0.05).

As expected, the OR rate was slower in the emulsions containing corn oil than in those without corn oil (Figs. 3 and 5), which can be attributed to the composition ripening mechanism discussed earlier. For glucose and maltose, there was a significant reduction in the OR rate when their concentration was raised from 0 to 5%, but then little changed when they were raised further, whereas for glycerol there was no significant change in the OR rate with additive concentration (Fig. 5). For both surfactants, the addition of the water-soluble additives did not cause a major change in the interfacial tension at the oil-water interface (Fig. 6). These results suggest that alterations in interfacial tension probably did not play a major role in impacting the OR rate. The trends in the data were fairly similar in the absence and presence of the corn oil, but the overall OR rate was less in its presence. Moreover, there was no correlation between the initial droplet size and polydispersity of the emulsions and their OR rates (Supplementary Fig. S8).

## Discussion

In the absence of corn oil, the droplet growth rate was higher for the Brij S20-stabilized emulsions than for the Tween 60-stabilized ones (Fig. 1a, c). We hypothesized that this effect was due to the presence of some surfactant micelles in the aqueous phase of the emulsions. The dependence of the initial droplet size on surfactant concentration was therefore measured for both emulsions (Supplementary Fig. S9). In general, the initial droplet size decreased with increasing surfactant concentration, because there was more surfactant available to cover a greater oil-water interfacial area. At a surfactant concentration of 0.1%, emulsions having a relatively small initial droplet diameter (<1 μm) could be fabricated. At surfactant concentrations greater than 0.3%, the initial mean droplet diameter did not decrease much with increasing surfactant concentration for both surfactants. These results suggest that emulsions fabricated with both Brij S20 and Tween 60 at a surfactant concentration of 0.5% may contain some non-adsorbed surfactant. These surfactants would be expected to self-assemble into micelles, which are small (typically < 20 nm) colloidal particles with a hydrophilic exterior and a hydrophobic core. Previous studies have shown that micelles can increase the OR rate of emulsions because they solubilize oil molecules within their hydrophobic interiors, thereby enhancing the mass transport rate^5,22^. According to the supplier, the critical micelle concentrations of Brij S20 and Tween 60 were 0.046 mM and 0.021 mM, respectively. Therefore, the concentration of micelles in the aqueous phase of the Tween 60-stabilized emulsions may be higher than those in the Brij S20-stabilized emulsions. Thus, the higher OR rate observed in the Brij S20-stabilized emulsions than in the Tween 60-stabilzed ones suggests that the micelle concentration in the aqueous phase was not the major factor influencing Ostwald ripening.

Another possible reason for the observed differences in the droplet growth rates between Brij S20-and Tween 60-stabilized emulsions is the structure of the hydrophilic heads at the oil-water interface. The properties of surfactants at interfaces are known to impact mass transport processes^23,24^. One would expect that the thicker and denser interfacial layer would slow down the movement of oil molecules, thereby inhibiting Ostwald ripening. Thus, the Brij S20-stabilized emulsions should be more resistant to Ostwald ripening than the Tween 60-stabilized because they would be expected to form thicker and denser interfaces. Again, this hypothesis was not supported by the experimental results, which suggested that the Tween 60-stabilized emulsions were actually more resistant to droplet growth in the absence of a ripening inhibitor (Fig. 1a, c).

We postulate that the apparent discrepancy between the predicted results and the actual results is due to droplet coalescence. Ostwald ripening occurs relatively rapidly in the absence of ripening inhibitors, leading to large oil droplets, which are known to be more susceptible to coalescence^2^. Moreover, during storage, large oil droplets cream to the top of emulsions due to the difference in density between the oil and water phases. In the cream layer, the oil droplets are closely packed together, which further increases their susceptibility to coalescence. Thus, if coalescence was prevalent in the emulsions used in this study, then the rate of droplet growth would not be correctly predicted by the OR theory. This effect would account for the fact that the Brij S20 had a lower interfacial tension than the Tween 60 (which should have been expected to decrease droplet growth through Ostwald ripening), but the rate of droplet growth was actually higher for Brij S20. A possible explanation for this phenomenon is that the interface formed by Brij S20 had less resistance to rupture than the one formed by Tween 60, and so the Brij S20 emulsions were more susceptible to droplet growth through coalescence, leading to a faster growth rate.

Mixing corn oil with *n*-decane alters the interfacial tension by altering the molecular composition and interactions at the oil-water interface^25^. When the oil phase of the emulsion consists solely of *n*-decane, the hydrophobic tails of surfactants interact only with the *n*-decane. However, if corn oil is mixed into the oil phase, the tails can interact with both *n*-decane and corn oil molecules, thereby altering the interfacial tension. Incorporating corn oil into the oil phase led to a slight decrease in interfacial tension for both surfactants (Fig. 2). Some molecules in corn oil have polar hydroxyl groups (including residual free fatty acids, monoglycerides, and diglycerides), which can interact with water slightly stronger than *n*-decane. Thus, the presence of corn oil can slightly reduce the interfacial tension. However, the magnitude of this effect was not sufficient to account for the dramatic decrease in droplet growth rate observed as the corn oil concentration inside the droplets was raised.

Interestingly, in the presence of the ripening inhibitor (corn oil), the rate of droplet growth was always less for the Brij S20-stabilized emulsions than for the Tween 60-stabilized ones (Fig. 1a, c). As mentioned earlier, in the absence of corn oil, we postulated that the rapid initial increase in droplet size due to Ostwald ripening led to some coalescence in the emulsions, as larger oil droplets are more susceptible to coalescence than smaller ones^2^. Thus, the rate of droplet growth did not follow the trends predicted by OR theory, such as an increase in OR rate with increasing interfacial tension (Eq. 1). In contrast, in the presence of the ripening inhibitor, the droplet growth rate is much lower, leading to the presence of smaller oil droplets. As a result, droplet growth due to coalescence is reduced. Consequently, the emulsions with the higher interfacial tension do undergo Oswald ripening more rapidly in this case, as predicted by OR theory.

Overall, these results indicate that corn oil was an effective ripening inhibitor for the *n*-decane emulsions, and that surfactant type did impact the rate of droplet growth to some extent. However, the pH of the aqueous phase did not strongly impact the droplet growth rate, which was mainly attributed to the fact that non-ionic surfactants were used.

The addition of water-soluble additives can alter the molecular organization of water molecules within the aqueous phase, thereby altering the equilibrium solubility of oil molecules. When hydrophobic substances (such as *n*-decane) are added to water, the energy required to break the hydrogen bonds between water molecules is not offset by the formation of new attractive interactions between hydrophobic substances and water molecules, indicating that this is energetically unfavorable. However, to compensate for the absence of the interactions between hydrophobic substances and water molecules, water molecules that surround hydrophobic substances rearrange themselves into a configuration that maximizes the hydrogen bonds between the water molecules. This results in the formation of a cage-like shell around the hydrophobic substances. According to previous studies^26–28^, the ability of glycerol to reduce water activity is greater than that of glucose and maltose, and the ability of glucose to reduce water activity is almost same to that of maltose. It indicated that glycerol could be bound to water molecules more tightly than glucose and maltose. Glucose, maltose, and glycerol were dissolved in the water at the same molar concentration, the value for the ratio of free water to the total water in glycerol solution should be always lower than those values for glucose and maltose solutions. Resultingly, the proportion of water-soluble substance/water attraction in all attractions (water-soluble substance-water and water-water attractions) in glycerol solution could be higher than glucose and maltose solutions. Therefore, it may be disadvantageous for the formation of a cage-like shell around *n*-decane molecules in glycerol solution compared with glucose and maltose solutions. Moreover, they may have altered the formation of the surfactant micelles in the aqueous phase, which could indirectly affect the OR rate. Thus, it is also possible that there were differences in the effects of glucose, maltose, and glycerol for these reasons.

As mentioned earlier, the propylene glycol behaved differently from the other water-soluble additives. Indeed, there was an increase in OR rate when the propylene glycol concentration increased above a particular value (Fig. 3g, h). This is attributed to the capacity of propylene glycol to alter the physicochemical properties of surfactants in aqueous solutions and emulsions. Propylene glycol has been reported to alter the solubility of surfactant monomers in aqueous solutions by modifying the strength of the hydrophobic effect^29,30^. The addition of propylene glycol to the aqueous phase has also been reported to improve the solubilization of limonene in microemulsions formed from non-ionic surfactants^31,32^ and to decrease the quantity of surfactant required for encapsulating limonene in a microemulsion-based delivery systems^33^. The optimum curvature, interfacial tension, and interfacial packing of surfactant monolayers can also be altered by proplylene glycol, as it alters the hydration of the hydrophilic head groups of surfactants^34,35^. It has also been reported that propylene glycol enhances the solubility of hydrophobic substances in water^36^. If propylene glycol simply increased the water-solubility of the *n*-decane, the OR rate of both emulsions stabilized by Tween 60 or Brij S20 would have increased. However, in practice, only the OR rate of the Brij S20-stabilized emulsion increased with increasing propylene glycol concentration (Fig. 3g, h). Moreover, despite the fact that the interfacial tension of the *n*-decane/Tween 60 solution was more affected by propylene glycol addition than the *n*-decane/Brij S20 solution (Fig. 4d), its impact on the OR rate was much more pronounced in the Brij S20-stabilized emulsions than in the Tween 60-stabilized emulsions (Fig. 3g, h). The impact of propylene glycol on the viscosity of the aqueous phase did not account for the observed differences in OR rates (Supplementary Fig. S6). It therefore appears that the interfacial layers formed by surfactants having a single hydrophilic chain are more susceptible to Ostwald ripening than the ones formed by surfactants having multiple hydrophilic chains. However, it is currently unclear how propylene glycol affects the characteristics of the interfacial layers when the structure of the hydrophilic head groups of surfactants changes significantly.

In the presence of the ripening inhibitor, increasing the propylene glycol concentration above a critical value (around 15%) led to a significant increase in the OR rate (Fig. 5g, h). Interestingly, although the addition of propylene glycol did not cause a major change in the interfacial tension, the OR rate increased with increasing propylene glycol concentration. As mentioned earlier, the properties of the interfacial layers may have been synergistically altered by both the presence of the corn oil and the propylene glycol, which reduced their resistance to Ostwald ripening. This combined effect did not appear to be significantly influenced by the structure of the surfactant used to create the emulsion.

In conclusion, this experiment showed that water-soluble additives effect the OR rate in oil-in-water emulsions by an amount that depends on their chemical structure and concentration, and the nature of the emulsifier used. Glucose, maltose, and glycerol addition inhibited the OR rate in the emulsions, whereas propylene glycol increased it. These effects were mainly attributed to the impact of these water-soluble additives on the interfacial tension and viscosity of the aqueous phase. All water-soluble additives caused either a slight decrease in the interfacial tension or had little effect on the interfacial tension, depending on surfactant type. This decrease in interfacial tension would be expected to reduce the driving force for Ostwald ripening, thereby slowing droplet growth. All additives increased the viscosity of the aqueous phase, which should slow the movement of oil molecules between the droplets, thereby reducing Ostwald ripening. These two effects would be expected to retard Ostwald ripening in the emulsions, which was true for the samples containing glucose, maltose, and glycerol but not for the ones containing propylene glycol. Our results may be important for formulating oil-in-water emulsions or nanoemulsions that are more resistant to Ostwald ripening, and therefore have a longer shelf life. This knowledge is especially important for systems where relatively polar oils are used, such as essential oils or flavor oils. Certain kinds of water-soluble additives can increase their stability, whereas others can reduce it. Consequently, it is important to select the most appropriate ones.

## Materials and methods

### Materials

Tween® 60 (Polyoxyethylene (20) sorbitan monostearate), Brij^®^ S20 (polyoxyethylene (20) stearyl ether), glycerol, and sodium azide were purchased from Sigma-Aldrich, Co. (St. Louis. MO, USA). *n*-Decane and propylene glycol were purchased from Daejung Chemicals and Metals (Siheung, Korea). Anhydrous glucose was purchased from Alfa Aesar (Ward Hill, MA, USA). Maltose monohydrate was purchased from Duksan General Science (Ansan, Korea). Corn oil was purchased from a local supermarket. All other chemicals were of reagent grade.

### Emulsion preparation

Initially, an aqueous phase was prepared by solubilizing 0.5% (*w*/*w*) Tween 60 or Brij S20 and 0, 5, 15, or 30% (*w*/*v*) of glycerol, propylene glycol, glucose, or maltose in phosphate buffer solution (10 mM, pH 7) containing 0.02% (*w*/*w*) sodium azide (as a non-food grade antimicrobial agent). Then, an oil phase was prepared by mixing 0, 5, 10, 20, or 30% (*w*/*w*) corn oil with *n*-decane. Coarse emulsions were then prepared by homogenizing the oil phase (5%, *w*/*w*) and aqueous phase (95%, *w/w*) for 2 min at 25 °C using a high-shear blender. Fine emulsions were then prepared by passing these coarse emulsions five times through a microfluidizer (MN400BF, Micronox, Seongnam, Korea) at 100 MPa. The pH of the final emulsions was then adjusted to 3 or 7. The emulsions were then stored at 25 °C in the dark and samples were collected periodically for analysis.

### Droplet size measurement

During storage, changes in the sizes of the droplets in the emulsions were measured using a laser diffraction particle size analyzer (BT-9300ST; Bettersize Instruments, Dandong, China). The emulsions were stirred for 2 min and then diluted using 10 mM phosphate buffer solution with the same pH as the emulsion prior to the droplet size measurements to prevent multiple scattering effects. During these measurements, continuous stirring was maintained to ensure sample homogeneity. The refractive index values of the oil and water phases were fixed at 1.411 and 1.333, respectively. The particle size data are reported as the volume-weighted mean diameter, ${d}_{43}=\sum {n}_{i}\cdot {d}_{i}^{4}/\sum {n}_{i}\cdot {d}_{i}^{3}$, where ${n}_{i}$ is the number of particles with diameter ${d}_{i}$.

The OR rates of the emulsions were calculated using Eq. 2, based on the Lifshitz-Slyozov-Wagner theory:

5 $${{r}_{t}}^{3}-{{r}_{0}}^{3}=\omega \cdot t$$

Here, *r*_0_ (μm) represents the radius of the oil droplet in the fresh emulsion, while *r*_t_ (μm) is the radius of the oil droplet in the emulsion after being stored for time ($t$; days). The OR rate (*ω*, μm³/day) was determined by performing a linear regression on the plot of (*r*_t_^3^ – *r*_0_^3^) against time. The relative OR rate was expressed as a percentage of the OR rate of the emulsion containing corn oil (or water-soluble substances) to the OR rate of the emulsion without corn oil (or water-soluble substances).

### Viscosity measurement

A sine wave vibro-viscometer (SV-10, A&D Company, Tokyo, Japan) outfitted with a 50 mL polycarbonate sample cup, the viscosity of the aqueous phases was determined. The sample cup was filled with 40 mL of the aqueous phase and the temperature was set at 25 °C. The driving electric current required to resonate two gold sensor plates at a frequency of 30 Hz was detected by this apparatus in order to determine the viscosity.

### Interfacial tension measurement

A DSA 25 pendant drop tensiometer (Krüss, Hamburg, Germany) was used to measure the interfacial tensions between *n*-decane and emulsifier solutions. Measurements were made at 25 °C. Oil (*n*-decane or the mixture of *n*-decane and corn oil) was placed A quartz cuvette, and a pendant drop was made in the oil using a NE45 needle with a 1.8 mm diameter. Using Advance 1.9.2.3. software, the interfacial tension values were calculated by fitting the profile of the pendant drop to its theoretical shape.

### Statistical analysis

From experiments done in triplicate, the values for means and standard deviations were obtained. Duncan’s multiple range test was run for mean comparison (*p* < 0.05) using IBM SPSS statistics version 21.0 (IBM, Armonk, NY, USA).

## Supplementary information

Supplemental Materials

## Data Availability

All data generated or analysed during this study are included in this published article and its supplementary information files.

## References

[1] Jie, Y. L. & Chen, F. S. Progress in the application of food-grade emulsions. *Foods* **11**, 2883 (2022). 36141011 10.3390/foods11182883 PMC9498284

[2] McClements, D. J. *Food Emulsions: Principles, Practices, and Techniques* 3rd edn (ed McClements, D. J.) 289–382 (CRC Press, 2015).

[3] Cai, Z. X. et al. Correlation between interfacial layer properties and physical stability of food emulsions: current trends, challenges, strategies, and further perspectives. *Adv. Colloid Interface Sci.* **313**, 102863 (2023). 36868168 10.1016/j.cis.2023.102863

[4] Tan, C. & McClements, D. J. Application of advanced emulsion technology in the food industry: a review and critical evaluation. *Foods* **10**, 812 (2021). 33918596 10.3390/foods10040812 PMC8068840

[5] Kabalnov, A. Ostwald ripening and related phenomena. *J. Dispers. Sci. Technol.* **22**, 1–12 (2001).

[6] Koroleva, M. Y. & Yurtov, E. V. Ostwald ripening in macro- and nanoemulsions. *Russ. Chem. Rev.* **90**, 293–323 (2021).

[7] Taylor, P. Ostwald ripening in emulsions. *Adv. Colloid Interface Sci.* **75**, 107–163 (1998). 10.1016/s0001-8686(03)00113-1 14672850

[8] Ryu, V., Corradini, M. G., McClements, D. J. & McLandsborough, L. Impact of ripening inhibitors on molecular transport of antimicrobial components from essential oil nanoemulsions. *J. Colloid Interface Sci.* **556**, 568–576 (2019). 31479830 10.1016/j.jcis.2019.08.059

[9] Trujillo-Cayado, L. A., Santos, J., Calero, N., Alfaro-Rodriguez, M. C. & Munoz, J. Strategies for reducing Ostwald ripening phenomenon in nanoemulsions based on thyme essential oil. *J. Sci. Food Agric.* **100**, 1671–1677 (2020). 31802496 10.1002/jsfa.10181

[10] Ariyaprakai, S., Hu, X. Y. & Tran, M. T. Spontaneous formation of flavor oil emulsions by using sucrose esters and emulsion stability study. *Food Biophys.* **14**, 41–48 (2019).

[11] Han, S. W., Song, H. Y., Moon, T. W. & Choi, S. J. Influence of emulsion interfacial membrane characteristics on Ostwald ripening in a model emulsion. *Food Chem.* **242**, 91–97 (2018). 29037741 10.1016/j.foodchem.2017.09.018

[12] Jang, Y., Park, J., Song, H. Y. & Choi, S. J. Ostwald ripening rate of orange oil emulsions: effects of molecular structure of emulsifiers and their oil composition. *J. Food Sci.* **84**, 440–447 (2019). 30714618 10.1111/1750-3841.14464

[13] Li, Y., Le Maux, S., Xiao, H. & McClements, D. J. Emulsion-based delivery systems for tributyrin, a potential colon cancer preventative agent. *J. Agric. Food Chem.* **57**, 9243–9249 (2009). 19731938 10.1021/jf901836f

[14] Raikos, V., Hayward, N., Hayes, H., Meroni, E. & Ranawana, V. Optimising the ratio of long- to short-chain triglycerides of the lipid phase to enhance physical stability and bioaccessibility of lycopene-loaded beverage emulsions. *Int. J. Food Sci. Technol.* **54**, 1355–1362 (2019).

[15] Choi, S. J. & McClements, D. J. Nanoemulsions as delivery systems for lipophilic nutraceuticals: strategies for improving their formulation, stability, functionality and bioavailability. *Food Sci. Biotechnol.* **29**, 149–168 (2020). 32064124 10.1007/s10068-019-00731-4 PMC6992823

[16] Kabalnov, A. S. & Shchukin, E. D. Ostwald ripening theory: applications to fluorocarbon emulsion stability. *Adv. Colloid Interface Sci.* **38**, 69–97 (1992).

[17] Lim, S. S. et al. Stabilization of orange oil-in-water emulsions: a new role for ester gum as an Ostwald ripening inhibitor. *Food Chem.* **128**, 1023–1028 (2011).

[18] Piorkowski, D. T. & McClements, D. J. Beverage emulsions: recent developments in formulation, production, and applications. *Food Hydrocoll.* **42**, 5–41 (2014).

[19] Molet-Rodríguez, A., Salvia-Trujillo, L. & Martín-Belloso, O. Beverage emulsions: key aspects of their formulation and physicochemical stability. *Beverages* **4**, 70 (2018).

[20] Ryu, V., McClements, D. J., Corradini, M. G. & McLandsborough, L. Effect of ripening inhibitor type on formation, stability, and antimicrobial activity of thyme oil nanoemulsion. *Food Chem.* **245**, 104–111 (2018). 29287320 10.1016/j.foodchem.2017.10.084

[21] Patel, H., Raval, G., Nazari, M. & Heerklotz, H. Effects of glycerol and urea on micellization, membrane partitioning and solubilization by a non-ionic surfactant. *Biophys. Chem.* **150**, 119–128 (2010). 20417021 10.1016/j.bpc.2010.03.015

[22] Weiss, J. & McClements, D. J. Mass transport phenomena in oil-in-water emulsions containing surfactant micelles: solubilization. *Langmuir* **16**, 5879–5883 (2000).

[23] Chen, Y., Narayan, S. & Dutcher, C. S. Phase-dependent surfactant transport on the microscale: Interfacial tension and droplet coalescence. *Langmuir* **36**, 14904–14923 (2020). 33269588 10.1021/acs.langmuir.0c02476

[24] Nowak, E., Kovalchuk, N. M., Che, Z. & Simmons, M. J. H. Effect of surfactant concentration and viscosity of outer phase during the coalescence of a surfactant-laden drop with a surfactant-free drop. *Colloid Surf. A-Physicochem. Eng. Asp.* **505**, 124–131 (2016).

[25] Kim, H. & Burgess, D. J. Prediction of interfacial tension between oil mixture and water. *J. Colloid Interface Sci.* **241**, 506–513 (2001).

[26] Alzamora, S. M., Chirife, J. & Gerschenson, L. N. Determination and correlation of the water activity of propylene glycol solutions. *Food Res. Int.* **27**, 65–67 (1994).

[27] Kerdudo, A., Fontaine-Vive, F., Dingas, A., Faure, C. & Fernandez, X. Optimization of cosmetic preservation: water activity reduction. *Int. J. Cosmet. Sci.* **37**, 31–40 (2015). 25256527 10.1111/ics.12164

[28] Wang, Q., Zhao, L., Li, C. & Cao, Z. The decisive role of free water in determining homogenous ice nucleation behavior of aqueous solutions. *Sci. Rep.* **6**, 26831 (2016). 27225427 10.1038/srep26831 PMC4881027

[29] Millard, J. W., Alvarez-Núñez, F. A. & Yalkowsky, S. H. Solubilization by cosolvents establishing useful constants for the log-linear model. *Int. J. Pharm.* **245**, 153–166 (2002). 12270252 10.1016/s0378-5173(02)00334-4

[30] Saberi, A. H., Fang, Y. & McClements, D. J. Fabrication of vitamin E-enriched nanoemulsions by spontaneous emulsification: Effect of propylene glycol and ethanol on formation, stability, and properties. *Food Res. Int.* **54**, 812–820 (2013).

[31] Garti, N., Yaghmur, A., Leser, M. E., Clement, V. & Watzke, H. J. Improved oil solubilization in oil/water food grade microemulsions in the presence of polyols and ethanol. *J. Agric. Food Chem.* **49**, 2552–2562 (2001). 11368635 10.1021/jf001390b

[32] Yaghmur, A., Aserin, A. & Garti, N. Phase behavior of microemulsions based on food-grade nonionic surfactants: effect of polyols and short-chain alcohols. *Colloid Surf. A-Physicochem. Eng. Asp.* **209**, 71–81 (2002).

[33] Fanun, M. Propylene glycol and ethoxylated surfactant effects on the phase behavior of water/sucrose stearate/oil system. *J. Dispers. Sci. Technol.* **28**, 1244–1253 (2007).

[34] Aramaki, K., Olsson, U., Yamaguchi, Y. & Kunieda, H. Effect of water-soluble alchols on surfactant aggregation in the C_12_EO_8_ system. *Langmuir* **15**, 6226–6232 (1999).

[35] Florence, A. T., Madsen, F. & Puisieux, F. Emulsion sabilization by non-ionic surfactants: the relevance of surfactant cloud point. *J. Pharm. Pharmacol.* **27**, 385–394 (1975). 237085 10.1111/j.2042-7158.1975.tb09466.x

[36] Strickley, R. G. Solubilizing exipients in oral and injectable formulations. *Pharm. Res.* **21**, 201–230 (2004). 15032302 10.1023/b:pham.0000016235.32639.23

