# Supplementary material for: Impact of hydrophilic substances on Ostwald ripening in emulsions stabilized by varied hydrophilic group surfactants
Source: NPJ Sci Food. 2024 Oct 5;8:76. doi: 10.1038/s41538-024-00316-4 (PMC11455911; doi:10.1038/s41538-024-00316-4)
Supplement: Supplementary file 1 — Supplemental Materials [file 41538_2024_316_MOESM1_ESM.docx]

Supplementary Fig. S1. Influence of the corn oil addition on droplet growth of *n*-decane-in-water emulsions.

**a** Emulsions prepared with pure *n*-decane. **b** Emulsions prepared with mixed oil (95% (*w*/*w*) *n*-decane and 5% (*w*/*w*) corn oil). **c** Emulsions prepared with mixed oil (90% (*w*/*w*) *n*-decane and 10% (*w*/*w*) corn oil). **d** emulsions prepared with mixed oil (80% (*w*/*w*) *n*-decane and 20% (*w*/*w*) corn oil). **e** Emulsions prepared with mixed oil (70% (*w*/*w*) *n*-decane and 30% (*w*/*w*) corn oil).

Supplementary Fig. S2. Ostwald ripening plots of the *n*-decane-in-water emulsions with the oil phase modified by the addition of corn oil.

**a** Brij S20-stabilized emulsions. **b** Tween 60-stabilized emulsions.

Supplementary Fig. S3. Influence of corn oil concentration in oil phase on the droplet size and polydispersity index of Brij S20- and Tween 60-stabilized emulsions freshly prepared at pH 3 and 7.

**a** Droplet size of emulsions freshly prepared at pH 3. **b** Polydispersity index of emulsions freshly prepared at pH 3. **c** Droplet size of emulsions freshly prepared at pH 7. **d** Polydispersity index of emulsions freshly prepared at pH 7. Values denoted by the different letters indicate the significant differences within emulsions prepared with the same emulsifier (*p* ≤ 0.05). Asterisk (*) indicates the significant difference between Brij S20- and Tween 60-stabilized emulsions containing the same amount of corn oil (*p* ≤ 0.05).

Supplementary Fig. S4. Ostwald ripening plots of the *n*-decane-in-water emulsions containing water-soluble substances.

**a** Ostwald ripening plots of Brij S20-stabilized emulsions prepared with glucose. **b** Ostwald ripening plots of Tween 60-stabilized emulsions prepared with glucose. **c** Ostwald ripening plots of Brij S20-stabilized emulsions prepared with maltose. **d** Ostwald ripening plots of Tween 60-stabilized emulsions prepared with maltose. **e** Ostwald ripening plots of Brij S20-stabilized emulsions prepared with glycerol. **f** Ostwald ripening plots of Tween 60-stabilized emulsions prepared with glycerol. **g** Ostwald ripening plots of Brij S20-stabilized emulsions prepared with propylene glycol. **h** Ostwald ripening plots of Tween 60-stabilized emulsions prepared with propylene glycol.

Supplementary Fig. S5. Influence of the water-soluble substances on the droplet size and polydispersity index of Brij S20- and Tween 60-stabilized emulsions prepared with pure *n*-decane.

**a** Droplet diameter of emulsions freshly prepared with glucose. **b** Polydispersity index of emulsions freshly prepared with glucose. **c** Droplet diameter of emulsions freshly prepared with maltose. **d** Polydispersity index of emulsions freshly prepared with maltose. **e** Droplet diameter of emulsions freshly prepared with glycerol. **f** Polydispersity index of emulsions freshly prepared with glycerol. **g** Droplet diameter of emulsions freshly prepared with propylene glycol. **h** Polydispersity index of emulsions freshly prepared with propylene glycol. Values denoted by the different letters indicate the significant differences within emulsions prepared with the same emulsifier (*p* ≤ 0.05). Asterisk (*) indicates the significant difference in values between Brij S20- and Tween 60-stabilized emulsions.

Supplementary Fig. S6. Influence of the addition of water-soluble substances on the viscosity of the aqueous phase.

Supplementary Fig. S7. Ostwald ripening plots of the oil (95% (*w*/*w*) *n*-decane and 5% (*w*/*w*) corn oil)-in-water emulsions containing water-soluble substances.

**a** Ostwald ripening plots of Brij S20-stabilized emulsions prepared with glucose. **b** Ostwald ripening plots of Tween 60-stabilized emulsions prepared with glucose. **c** Ostwald ripening plots of Brij S20-stabilized emulsions prepared with maltose. **d** Ostwald ripening plots of Tween 60-stabilized emulsions prepared with maltose. **e** Ostwald ripening plots of Brij S20-stabilized emulsions prepared with glycerol. **f** Ostwald ripening plots of Tween 60-stabilized emulsions prepared with glycerol. **g** Ostwald ripening plots of Brij S20-stabilized emulsions prepared with propylene glycol. **h** Ostwald ripening plots of Tween 60-stabilized emulsions prepared with propylene glycol.

Supplementary Fig. S8. Influence of the water-soluble substances on the droplet size and polydispersity index of Brij S20- and Tween 60-stabilized emulsions prepared with the mixture of *n*-decane (95% (*w*/*w*)) and corn oil (5% (*w*/*w*)).

**a** Droplet diameter of emulsions freshly prepared with glucose. **b** Polydispersity index of emulsions freshly prepared with glucose. **c** Droplet diameter of emulsions freshly prepared with maltose. **d** Polydispersity index of emulsions freshly prepared with maltose. **e** Droplet diameter of emulsions freshly prepared with glycerol. **f** Polydispersity index of emulsions freshly prepared with glycerol. **g** Droplet diameter of emulsions freshly prepared with propylene glycol. **h** Polydispersity index of emulsions freshly prepared with propylene glycol. Values denoted by the different letters indicate the significant differences within emulsions prepared with the same emulsifier (*p* ≤ 0.05). Asterisk (*) indicates the significant difference in values between Brij S20- and Tween 60-stabilized emulsions.

Supplementary Fig. S9. Influence of emulsifier concentration on the initial droplet size of *n*-decane-in-water emulsion (5% (*w*/*w*)) at pH 7.
